# Altered hippocampal microstructure and function in children who experienced Hurricane Irma

**DOI:** 10.1002/dev.22071

**Published:** 2020-12-16

**Authors:** May I. Conley, Lena J. Skalaban, Kristina M. Rapuano, Raul Gonzalez, Angela R. Laird, Anthony Steven Dick, Matthew T. Sutherland, Richard Watts, B.J. Casey

**Affiliations:** 1 Department of Psychology, Yale University, New Haven, CT, USA; 2 Department of Psychology, Florida International University, Miami, FL, USA; 3 Department of Physics, Florida International University, Miami, FL, USA

**Keywords:** development, hippocampus, memory, neurogenesis, restriction spectrum imaging, stress

## Abstract

Hurricane Irma was the most powerful Atlantic hurricane in recorded history, displacing 6 million and killing over 120 people in the state of Florida alone. Unpredictable disasters like Irma are associated with poor cognitive and health outcomes that can disproportionately impact children. This study examined the effects of Hurricane Irma on the hippocampus and memory processes previously related to unpredictable stress. We used an innovative application of an advanced diffusion-weighted imaging technique, restriction spectrum imaging (RSI), to characterize hippocampal microstructure (i.e., cell density) in 9- to 10-year-old children who were exposed to Hurricane Irma relative to a non-exposed control group (i.e., assessed the year before Hurricane Irma). We tested the hypotheses that the experience of Hurricane Irma would be associated with decreases in: (a) hippocampal cellularity (e.g., neurogenesis), based on known associations between unpredictable stress and hippocampal alterations; and (b) hippocampal-related memory function as indexed by delayed recall. We show an association between decreased hippocampal cellularity and delayed recall memory in children who experienced Hurricane Irma relative to those who did not. These findings suggest an important role of RSI for assessing subtle microstructural changes related to functionally significant changes in the developing brain in response to environmental events.

## 1 | INTRODUCTION

As Hurricane Irma, the most powerful Atlantic hurricane in recorded history, moved toward the southern coast of Florida in September 2017, over 6 million Florida residents were evacuated from their homes leading to food, water, and fuel shortages throughout the state (National Weather Service, 2018). Irma made landfall as a Saffir-Simpson Category 4 hurricane, ripping down power lines, and leaving two-thirds of individuals and families in Florida without power as it tore roofs off their homes and flooded their streets. Ultimately, Irma left a death toll of over 120 in its wake in Florida alone (Issa et al., 2018).

A large epidemiological literature associates exposure to disasters with poor mental and physical health (Furr et al., 2010; Galea et al., 2005; Garrison et al., 1995; Neria et al., 2008; Rubonis & Bickman, 1991) and poor cognitive outcomes (Bahrick et al., 1998; Brandes et al., 2002; Hikichi et al., 2017; Yasik et al., 2007). More specifically, time-marked, unpredictable events such as hurricanes and other natural disasters have been linked to alterations in brain and behavior (Chen et al., 2019; Ke et al., 2018; Kessel et al., 2018; Kopala-Sibley et al., 2016), with some evidence suggesting that children are disproportionately affected by natural disasters relative to adults (Satcher et al., 2007). Notably, prior investigations of neural mechanisms impacted by other forms of unpredictable stress provide important insight into how unpredictable events can lead to lasting or transitory alterations in brain and behavior.

The experience and expression of emotions related to environmental events is associated with the limbic system (Blum et al., 2000). One limbic region known to be impacted by unpredictable stress in the hippocampus (Cameron & Schoenfeld, 2018; McEwen et al., 2015; Redish, 2016), which plays a key role in learning and memory (Cohen & Eichenbaum, 1993; O’Keefe & Nadel, 1978; Scoville & Milner, 1957; Squire, 2009) about emotional events and places (Girardeau et al., 2017; Kensinger & Corkin, 2004; LeDoux, 1993; Phelps, 2004). The duration, intensity, and predictability of stressful events (Joëls & Baram, 2009; Tottenham & Sheridan, 2010) can differentially impact hippocampal structure and function at microstructural (i.e., cellular) and macrostructural levels (Chen et al., 2010; Liston & Gan, 2011; Lupien et al., 2009; McEwen, 1999, 2007). Hurricanes like Irma vary in their trajectory, duration, and intensity of destruction, often bringing about unpredictable events including evacuation, floods, power outages, and food and water shortages, which can increase hurricane-related stress and vulnerability for long-term mental health problems (McLaughlin et al., 2010). Time-limited stressors can inhibit the induction of long-term potentiation (LTP) in the hippocampus (Diamond et al., 1990; Foy et al., 1987), with predictability modulating the magnitude of LTP (Kavushansky et al., 2006; Shors et al., 1989, 1990). LTP is a reflection of synaptic plasticity and associated with dendritic arborization and the formation of new synapses (Bliss & Gardner-Medwin, 1973; Bliss & Lømo, 1973). Hippocampal neurogenesis is also linked to neural plasticity and together these processes are thought to support optimal exploration of novel events and environments (Glasper et al., 2012). Unpredictable stressful events are associated with hippocampal microstructure changes in adult animal models including reductions in hippocampal neurogenesis (Gould et al., 1997, 1998; Tanapat et al., 2001), spine density in basal dendrites of CA1 (Diamond et al., 2006), and apical dendrites of CA3 (Chen et al., 2008, 2010; Magariños & McEwen, 1995; Stewart et al., 2005). Importantly, for the purposes of this study, alterations in the hippocampus to unpredictable events are observed in younger non-human animals (Hollis et al., 2013; Romeo, 2017; Simon et al., 2005; Tanapat et al., 1998). Specifically, pre-pubertal and adolescent non-human animals exhibit protracted stress-responses relative to adults (Lupien et al., 2009; Romeo, 2017). While these long lasting changes may be associated with elevated risk for disease (Kim et al., 2015), dendritic atrophy and decreases in hippocampal neurogenesis may facilitate adaptive responding and focus on a stressor by mitigating structural changes and increasing temporary vigilance in the face of uncertain environmental conditions (Cameron & Schoenfeld, 2018). Together this work highlights the responsivity of the hippocampus to unpredictable experiences. However, less is known about microstructural changes that may underlie these associations in the developing human brain underscoring the importance of elucidating behaviorally significant changes in the hippocampus following natural disasters like Irma.

**FIGURE 1:**
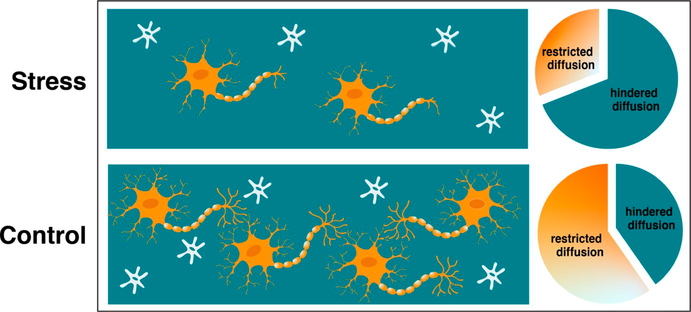
Restriction spectrum imaging schematic. Intracellular water diffusion within cells (neurons in orange; glial cells in gray) is restricted, while extracellular water diffusion (teal) is hindered. Stress-related alterations in the hippocampus (e.g., decreases in neurogenesis, dendritic atrophy) would be associated with a decrease in restricted (intracellular) diffusion (top panel) relative to control conditions (i.e., no acute stress; typical neurogenesis) (bottom panel)

Recent advances in diffusion-weighted imaging (DWI) allow for non-invasively examining microstructural detail of the hippocampus. For example, changes in hippocampal microstructure (e.g., neurite density and dispersion) measured with advanced DWI techniques are associated with age-related changes in memory performance in adults (Radhakrishnan et al., 2020; Zhang et al., 2012), demonstrating that probing the microstructure of gray matter may be important for characterizing functionally relevant changes. Other recent methodological advances using diffusion magnetic resonance imaging provide further opportunity to examine the microstructure of deep gray matter by providing estimates of cell and neurite density. Restriction spectrum imaging (RSI) provides an estimate of the cellularity of gray-matter tissue in specific areas of the brain by separating the diffusion-weighted MRI signal into restricted and hindered diffusion components, which represent intracellular and extracellular water signal, respectively (White, Leergaard, et al., 2013; White, McDonald, et al., 2013; White et al., 2014; Figure 1). Accordingly, a greater fraction of restricted component (i.e., a greater proportion of restricted motion of water molecules) is believed to indicate greater tissue cellularity, an interpretation which has been histologically confirmed (White, Leergaard, et al., 2013). This methodology provides a novel opportunity to investigate microstructural changes in the developing human brain following exposure to unpredictable and/or stressful events.

Here, we utilized RSI to test for microstructural differences in the hippocampus of children exposed to Hurricane Irma, a naturalistic, unpredictable event. More specifically, we evaluated whether Irma-exposed children, tested in the year prior to Hurricane Irma, show decreased hippocampal cellularity relative to a non-exposed control group, tested during the year before Hurricane Irma. To test for regional specificity of any hippocampal effects, we examined all subcortical brain regions. To evaluate functional consequences of Irma and changes in hippocampal cellularity between Irma-exposed and non-exposed groups, we tested whether Irma-exposure was associated with group differences in hippocampal-related memory (i.e., delayed recall). We hypothesized that the experience of Hurricane Irma would be associated with decreases in (a) hippocampal cell density and (b) delayed recall memory.

## 2 | METHODS

### 2.1 | Participants

**FIGURE 2:**
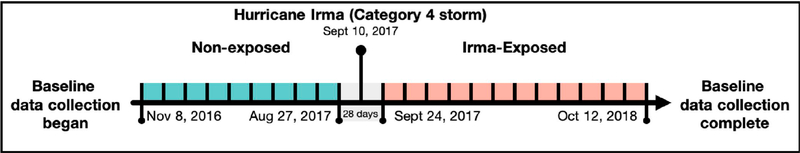
Experimental design. Participants from the same geographic location were divided into two groups based on when Adolescent Brain Cognitive Development baseline assessments and magnetic resonance imaging scans were acquired. The Irma non-exposed group was scanned during the year prior to Hurricane Irma. The Irma-exposed group was scanned during the year following Hurricane Irma

Participants were 9- and 10-year-old children from the South Florida site of the Adolescent Brain Cognitive Development (ABCD) Study® (https://ABCDStudy.org, https://nda.nih.gov/abcd; Volkow et al., 2018) included in the 2.0.1 ABCD Data Release (https://doi.org/10.15154/1504041). Detailed descriptions of ABCD Study® sampling procedures and design are detailed elsewhere (Compton et al., 2019; Garavan et al., 2018). To assess whether Hurricane Irma exposure was related to hippocampal microstructure and memory, participants were divided into non-exposed and Irma-exposed groups based on scan date. Although Hurricane Irma made landfall in Florida on September 10, 2017, National Hurricane Center monitoring and media coverage began on August 26, 2017 (14 days prior; Blake, 2017). Because media coverage of disasters and stressful events has been associated with acute stress and later PTSD (Aber et al., 2004; Gil-Rivas et al., 2007; Pfefferbaum et al., 2001, 2003; Thompson et al., 2019), ABCD participants tested between August 26 and September 10 were excluded to avoid anticipatory effects of the hurricane resulting from news sources (Figure 2). Because the non-exposed group was slightly older (mean age in months = 119.42 (*SD* = 7.18)) than the Irma-exposed group (mean age in months = 117.31 (*SD* = 7.52)) (*t* = 3.14, *df* = 424.65, *p* < .01) and there are known associations between typical development and hippocampal neurogenesis (He & Crews, 2007; Hodes et al., 2009), 25 (of 53) of the youngest participants (ages 108–109 months) were randomly excluded from the South Florida Irma-exposed group to eliminate significant differences in age, and age (in addition to pubertal status) was included as a control variable in all models.^1^

The South Florida, Irma-exposed group was comprised of 232 children (52% Girls; 69.4% pre- or early-pubertal^2^; 66% Hispanic; 21% Black; 8% White; 5% Other) (Figure S1). The South Florida non-exposed group was comprised of 191 children (45% Girls; 62.9% pre- or early-pubertal; 79% Hispanic; 10% Black; 8% White; 2% Asian; 1% Other). Exclusionary criteria for this study included a diagnosis of autism spectrum disorder (*n* = 4), history of epilepsy or seizures (*n* = 5), and missing demographic (*n* = 76), Rey Auditory Verbal Learning Test (RAVLT) (*n* = 23), or imaging data (*n* = 62). Individuals with outlier values (1.5 × IQR) in the RSI data were also excluded (*n* = 47).

Our primary hypotheses focused on differences between non-exposed and Irma-exposed groups from the same geographical location and social structure (i.e., South Florida) that were group matched on gender, pubertal status, age, and family income. However, to assess for specificity of the results to individuals proximal to Hurricane Irma, ancillary analyses considered differences between groups tested in the year prior to and following Hurricane Irma from a distal, non-exposed Northeastern ABCD site using a similar sample size and an identical scanner platform with the same DWI sequences. The operational definition of pre- and post-Irma groups was identical to the South Florida non-exposed and Irma-exposed groups (i.e., children scanned prior to August 26 and after September 10; Figure S2), however, geographic location, pubertal status, family income, parent education level, and racial and ethnic diversity varied significantly between these proximal and distal sites (see Figure S3 for distribution of demographic variables across sites).

The distal, Northeastern pre-Irma group was comprised of 181 children (44.1% Girls; 80.9% pre- or early-pubertal; 20.0% Hispanic; 9.2% Black; 61.0% White; 3.6% Asian; 6.2% Other). The distal, Northeastern post-Irma group was comprised of 195 children (48.1% Girls; 68.0% pre- or early-pubertal; 24.9% Hispanic; 20.4% Black; 45.3% White; 9.4% Other).

### 2.2 | Neuroimaging data collection

The ABCD scanning protocol includes 3D T1- and T2-weighted images, diffusion-weighted images, and resting-state and task-based function MRI measures previously detailed in Casey et al. (2018). Data were collected on a 3 Tesla Siemens MAGNETOM Prisma scanner with a 32-channel head coil. Diffusion images were collected using a spin-echo EPI acquisition with the following parameters: TR = 88 ms, TE = 4,100 ms, flip angle = 90°, 81 slices, voxel size = 1.7 mm^3^, multiband slice acceleration factor = 3, 7 *b* = 0 s/mm^2^ frames, and 6 directions at *b* = 500 s/mm^2^, 15 directions at *b* = 1,000 s/mm^2^, 15 directions at *b* = 2,000 s/mm^2^, and 60 directions at *b* = 3,000 s/mm^2^.

### 2.3 | Image preprocessing, RSI, and volumetric data

Diffusion magnetic resonance imaging and structural magnetic resonance imaging (sMRI) data were processed by the ABCD Study Data Analysis, Informatics and Resources Center using methods previously detailed in Hagler et al. (2019). Restricted normalized isotropic (N0) metrics were calculated for subcortical gray matter using a linear estimation approach (White, Leergaard, et al., 2013; White, McDonald, et al., 2013; White et al., 2014) with atlas-based segmentation (Fischl et al., 2002). Hippocampal volume differences were tested using data computed from the pre-processed T1 images using FreeSurfer v5.3 and labeled using an atlas-based volumetric segmentation procedure (Fischl et al., 2002; Hagler et al., 2019).

### 2.4 | Hippocampal-related behavioral function

The RAVLT is a widely used and robust measure of auditory learning, memory, and recall (Lezak et al., 2004; Luciana et al., 2018). The test involves five learning trials of 15 unrelated words (list A). After each trial, participants are asked to recall as many words as possible. After the initial five learning trials, participants are presented with a distractor list of 15 new words (list B) and are then asked to recall as many words as possible from the new list (list B). Next, an immediate recall trial is assessed for words from the initial list (list A). After a 30-min delay (where participants complete other non-verbal tasks or rest), a final delayed recall trial is assessed for words from the initial list (list A). Previous work has established the RAVLT as a reliable measure of hippocampal integrity (Saury & Emanuelson, 2017) and hippocampal-dependent memory (Stevenson et al., 2018), linking the delayed recall trial in particular to hippocampal function (Wolk et al., 2011). Here, we assessed a behavioral correlate of hippocampal function using performance (total correct) on the delayed recall trial (i.e., RAVLT Trial VII).

### 2.5 | Analytic approach

Analyses were performed in R version 3.6.3 (R Core Team, 2020) using the *gamm4* package (Wood & Scheip, 2020). Mixed-effect models were used to evaluate RSI measures in subcortical regions as well as verbal memory between non-exposed and Irma-exposed groups. For all models, covariates included fixed effects for gender, interview age, race/ethnicity, parental education, and household income, and a random effect for family ID. In addition, RSI models also included intracranial volume, motion, pubertal development, and the interaction of gender and pubertal development as fixed covariates. Supplemental analyses restricting the sample to only pre- and early-pubertal participants were conducted (Supplemental Analyses 1) in addition to supplemental analyses including trauma history, threat exposure, and history of anxiety disorders and PTSD as other fixed covariates (Supplemental Analyses 2). All analyses were Bonferroni corrected for multiple comparisons. Non-parametric significance was assessed using permutation testing by randomly shuffling data 10,000 times. Non-parametric *p*-values were computed by dividing the number of times the randomly permuted *t*-statistic was greater than the observed *t*-statistic by the number of tests performed (i.e., # observations > |*t*|/10,000 + 1). Additionally, because there were no a priori hypotheses that effects would be lateralized, RSI was averaged between hemispheres for each subcortical region. Ancillary analyses applied identical models to the distal, Northeastern sample to evaluate if any of the findings detected in the South Florida sample could be attributed to other background cohort characteristics (e.g., sampling protocols, other overlooked events occurring in September 2017).

Following the initial region of interest (ROI) analyses, a *post hoc* analysis of voxel-wise data was performed to further examine the spatial specificity of our imaging results. Post hoc analyses were performed by applying the same model from the ROI analyses to every voxel within the subcortex utilizing the *cifti* (Muschelli, n.d.) and *lme4* (Bates et al., 2015) packages. Given previous work showing that the effects of stress can impact different subfields of the hippocampus (Hawley & Leasure, 2012; McEwen et al., 2015), the distribution of voxel-wise RSI (i.e., restricted diffusion) values was plotted as a function of the anterior/posterior coordinate axis (i.e., *y*-axis coordinates) to further evaluate spatial specificity within the hippocampus.

## 3 | RESULTS

### 3.1 | Decreased hippocampal cellularity in Irma-exposed group

Although a substantial literature across the fields of neuroscience and psychology describes relationships between unpredictable events and macroscale changes in hippocampal structure and function, less is known about how these associations emerge in the developing human brain. Thus, the primary aim of the current study was to determine if RSI could be used to detect subtle microstructural differences in the hippocampus of children exposed to a naturalistic unpredictable event, Hurricane Irma (hypothesis 1).

**FIGURE 3:**
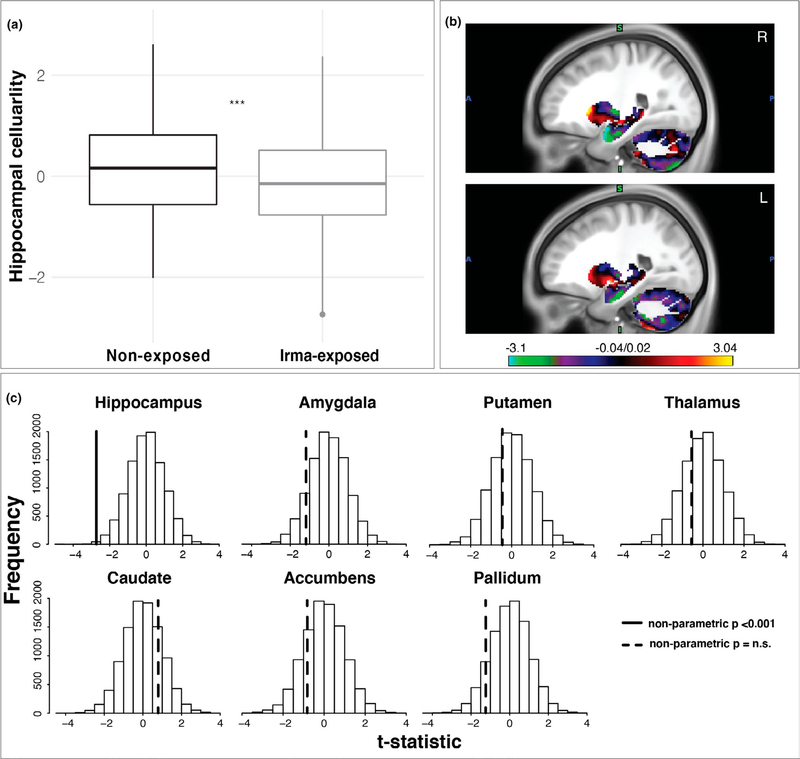
Differences in restriction spectrum imaging-based hippocampal cellularity among South Florida Irma-exposed relative to non-exposed children. (a) Irma exposure was related to decreased hippocampal cell density; (b) *post hoc* voxel-wise analysis demonstrated spatial specificity of findings to the hippocampus in right and left hemispheres; (c) results of permutation testing (10,000 + 1 iterations) demonstrated specificity of Irma exposure on hippocampal cellularity and not to other subcortical regions. The histograms show the null distribution of *t*-statistics with observed effects indicated with vertical lines (solid lines represent non-parametric significance and dashed lines represent non-parametric non-significance)

Consistent with hypothesis 1, and with previous literature linking unpredictable stress to dendritic atrophy and decreased neurogenesis in the hippocampus, lower restricted diffusion signal, consistent with lower cellularity, was observed in the hippocampus of the South Florida Irma-exposed group relative to the South Florida non-exposed control group (*β* = −3.90 × 10^−3^ (*SE* = 1.35 × 10^−3^), *t* = −2.875, *p* = .004, *r*^*2*^ (adj) = .05, Δ*r*^*2*^ (adj) = .02; Figure 3a).^3,4^ Control analyses demonstrated that these findings are robust to the impact of puberty and various adverse life events or experiences on hippocampal microstructure and are described in supplemental materials. Next, we used permutation testing to evaluate the specificity of this effect to the hippocampus by testing all subcortical regions. Across all subcortical areas, only the hippocampus showed significant differences in cellularity between the Irma-exposed and non-exposed control group (Figure 3c).

**FIGURE 4:**
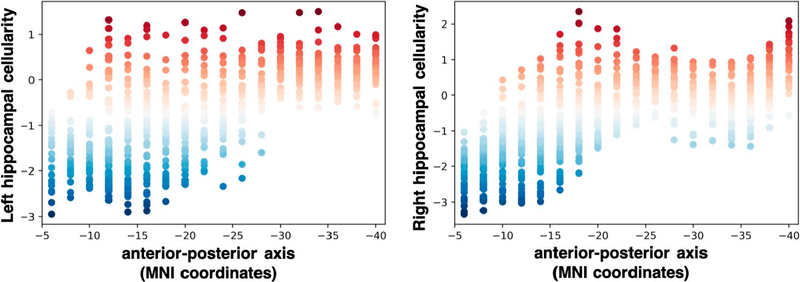
Group differences in hippocampal cellularity are predominantly concentrated in anterior hippocampus. The distribution of restriction spectrum imaging values (i.e., restricted diffusion) derived from a post hoc voxel-wise analysis based on the Fischl atlas (Fischl et al., 2002) revealed decreases in left and right hippocampal cellularity in the Irma-exposed relative to non-exposed group are more concentrated in anterior hippocampus

A *post hoc* voxel-wise analysis of deep gray matter further revealed the spatial specificity of these findings. Consistent with the ROI results above, voxel-wise models revealed a robust relationship between cellularity measured with RSI and Irma exposure in the hippocampus (Figure 3b). Further evaluation of the spatial distribution of differences in hippocampal cellularity between the Irma-exposed and non-exposed groups revealed that this effect was more concentrated in anterior hippocampus (Figure 4). There was no significant difference in hippocampal volume between Irma-exposed and non-exposed groups (*β* = 12.5 (*SE* = 32.2), *t* = 0.389, *p* = .70, *r*^*2*^ (adj) = .38, Δ*r*^*2*^ (adj) < .001). Together these results are consistent with the hypothesis that decreased hippocampal cellularity is associated with exposure to Hurricane Irma.

### 3.2 | Decreased hippocampal-related memory in Irma-exposed group

**FIGURE 5:**
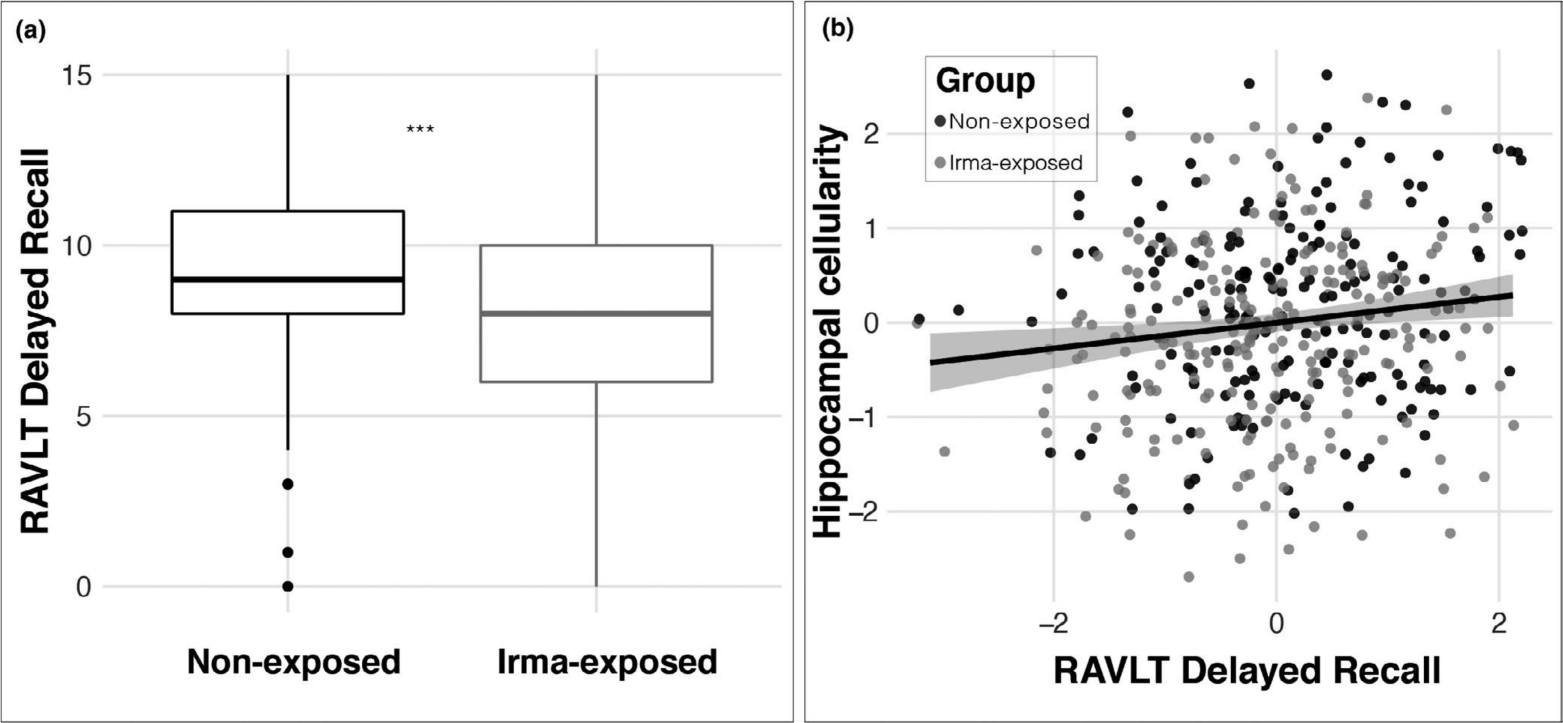
Differences in hippocampal function in Irma-exposed and non-exposed children. (a) Irma exposure was associated with poorer delayed recall; (b) less RSI-based hippocampal cellularity was associated with poorer delayed recall memory. RAVLT: Rey Auditory Verbal Learning Test; RSI, restriction spectrum imaging

To assess the behavioral implications of any differences observed in hippocampal cellularity between the Irma-exposed and non-exposed groups, we evaluated memory performance based on previous work showing associations between delayed recall and hippocampal function (Wolk et al., 2011). Consistent with hypothesis 2, this analysis showed decreased performance in the South Florida Irma-exposed group relative to the non-exposed control group (*β* = −0.71 (*SE* = 0.28), *t* = −2.511, *p* = .01, *r*^*2*^(adj) = .08, Δ*r*^*2*^ (adj) = .01; Figure 5a).^5^ An ancillary analysis confirmed that this relationship was robust even when controlling for fluid intelligence (*β* = −0.67 (*SE* = 0.28), *t* = −2.402, *p* = .01, *r*^*2*^ (adj) = .12, Δ*r*^*2*^ (adj) = .01). Furthermore, delayed recall was associated with hippocampal cell density (*β* = 24.53 (*SE* = 10.63), *t* = 2.307, *p* = .02, *r*^*2*^ (adj) = .07, Δ*r*^*2*^ (adj) = .01) such that children with lower hippocampal cellularity showed poorer delayed recall (Figure 5b).

### 3.3 | Distal comparison groups show no differences in hippocampal cellularity or delayed recall

**FIGURE 6:**
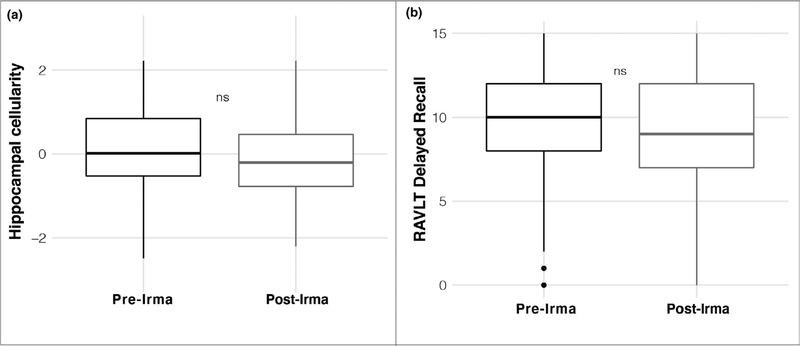
The distal, non-exposed Northeastern site revealed no group differences in (a) hippocampal cellularity; or (b) delayed recall between Pre- and Post-Irma groups. RAVLT, Rey Auditory Verbal Learning Test

To assess for specificity of results to individuals proximal to Hurricane Irma, ancillary analyses considered differences between individuals tested in the year prior to and following Hurricane Irma from a distal, non-exposed Northeastern ABCD site. This analysis revealed no significant difference in RSI-based hippocampal cellularity between groups scanned prior to and following Irma from the distal, Irma non-exposed Northeastern site (*β* = −1.67 × 10^−3^ (*SE* = 1.31 × 10^−3^), *t* = 1.28, *p* = .20, *r*^*2*^ (adj) = .03, Δ*r*^*2*^ (adj) < .001; Figure 6a). Additionally, this comparison analysis revealed no significant difference in delayed recall between groups tested prior to and following Irma from the distal, non-exposed Northeastern site (*β* = 0.06 (*SE* = 0.35), *t* = 0.187, *p* = .85, *r*^*2*^ (adj) = .05, Δ*r*^*2*^ (adj) < .001; Figure 6b).

## 4 | DISCUSSION

The current study examined whether the unpredictable and uncontrollable events of Hurricane Irma were associated with neural and behavioral signatures in children. Given that unpredictable stressful events have been associated with cellular alterations in the hippocampus, we employed a novel application of RSI to characterize hippocampal microstructural changes (i.e., decreases in cellularity) in response to a naturalistic, unpredictable, and stressful event. The results showed an association between exposure to Hurricane Irma and decreased RSI-based hippocampal cellularity in 9- and 10-year-old children. Analyses across all subcortical regions revealed specificity of this finding to the hippocampus and not to other subcortical regions, demonstrating the sensitivity of RSI to detect changes in hippocampal microstructure as a function of environmental experiences. The behavioral significance of these findings was supported by significantly poorer delayed recall performance in children exposed to Irma compared to non-exposed children, which was associated with less hippocampal cellularity. These findings highlight an important role of RSI in detecting meaningful, yet subtle microstructure changes in the hippocampus in response to an unpredictable event during development.

Our results are consistent with elegant animal work showing dendritic atrophy (Chen et al., 2008, 2010; Eiland et al., 2012; Eiland & Romeo, 2013; Magariños & McEwen, 1995; Stewart et al., 2005) and neurogenesis reductions (Gould et al., 1997, 1998; Tanapat et al., 2001) in the hippocampus following exposure to unpredictable stress. This work suggests that hippocampal microstructural differences between Irma-exposed and non-exposed children may be related to these previously established cellular alterations following stress. Additionally, the spatial distribution of cellularity differences associated with Hurricane Irma demonstrated specificity in the anterior hippocampus relative to the posterior hippocampus. This finding is consistent with previous animal work demonstrating decreases in ventral (analogous to anterior in primates; Fanselow & Dong, 2010) hippocampal neurogenesis following unpredictable stress (Hawley & Leasure, 2012; Hawley et al., 2012; Maggio & Segal, 2007). Although much remains to be learned about the role of neurogenesis and dendritic atrophy in neurobiological and behavioral responses to unpredictable events, previous studies posit that these stress-related alterations may serve to temporarily increase cautiousness and focus on stressors (Cameron & Schoenfeld, 2018; Schoenfeld et al., 2017). For example, memory impairment following stress (Chen et al., 2010; de Quervain et al., 1998; Logue et al., 2018) may serve to promote discernment between stress-relevant and -irrelevant information (Oitzl et al., 2010). In the face of a hurricane, for example, a heightened focus on novel, relevant information may facilitate quick responding in preparation for evacuation. Previous animal work shows that the magnitude of memory deficit following brief, acute stress correlates with reduced density of apical dendritic spines (Chen et al., 2010), suggesting that alterations in hippocampal microstructure may be involved in adaptive responding to environmental stressors. We found that Irma exposure was related to lower delayed recall performance, and that hippocampal cellularity was significantly associated with delayed recall in children, which raises the possibility that changes in hippocampal microstructure detected with RSI may relate to less processing of irrelevant information when adapting to a real-world stressful event (i.e., Hurricane Irma). Alternatively, these changes may be associated with long-term poor outcomes in children following a natural disaster as suggested by Satcher et al. (2007). Further research is needed to understand the temporal dynamics of changes in hippocampal microstructure and how they relate to outcomes, and whether these changes are stable over time.

The current findings advance our current understanding of microstructural changes related to unpredictable events and hippocampal-related memory function; however, limitations of observational approaches must be considered. First, although a substantial literature links hurricane exposure to stress (Garrison et al., 1995; Neria et al., 2008), the baseline ABCD study data do not include a validated measure of self-perceived stress. In addition, psychopathology, social support, and past trauma exposure or other hardships such as financial burden or limited access to resources (Layne et al., 2014) can moderate the effects of unpredictable events on perceived stress (Furr et al., 2010). It is also likely that participants in the South Florida non-exposed control group experienced varying levels of adversity at other times. However, Supplemental Analyses 2 showed that our findings were robust even when controlling for parent-reported trauma history, threat exposure, and clinical disorders such as anxiety and PTSD. Future work that incorporates measures of self-perceived stress and other important moderators and mediators such as family relationships or parental care (Callaghan & Tottenham, 2016) is needed to further inform the specificity of the effect of unpredictable stress on hippocampal microstructure and function. Although the current study suggests an association between hippocampal cellularity and long-term memory, we are unable to relate these changes in memory to specific details about Irma because the ABCD study, on which this study is based, does not evaluate memory details for Irma-related events. Second, while Hurricane Irma provided an opportunity to evaluate the impact of a naturalistic unpredictable event on subcortical microstructure, the South Florida subsample of the ABCD cohort does not reflect the geographic, demographic, or socioeconomic distribution of children across the United States or world (Compton et al., 2019; Garavan et al., 2018) and it is unclear whether our findings would generalize to other populations. Moreover, all participants were 9- and 10-yearolds and previous work suggests that changes in neurodevelopment with age and puberty can interact with the effects of stress (Gee and Casey, 2015; Gunnar & Quevedo, 2007; Lupien et al., 2009; Romeo, 2017). For example, the hippocampus continues to develop throughout adolescence (Anderson & Teicher, 2004; Benes et al., 1994; Giedd et al., 1996; Giedd & Rapoport, 2010; Meyer & Ferres-Torres, 1978) when the brain may be more sensitive to glucocorticoids (Lee et al., 2003) and when gender differences in stress-response and stress-related outcomes such as depression and anxiety emerge (Leussis & Andersen, 2008). Given previous work indicating that the effects of stress during adolescence may be longer-lasting relative to adults (McCormick et al., 2010; Romeo, 2018), it will be important to assess potential sensitive periods of greater risk or resilience throughout development. Similarly, our analyses are cross-sectional and cannot address causality. It is possible that differences in hippocampal microstructure between Irma-exposed and non-exposed children could be attributed to other background factors. Our ancillary analyses of a distal, non-exposed Northeastern site that showed no differences between children assessed the year prior to and following Hurricane Irma provide some confidence of the specificity of the results to Irma exposure. However, future longitudinal analyses and studies with optimized measures of stressor timing, type of stress, and cumulative stress will be needed for understanding how changes in subcortical microstructure emerge and relate to previously established interactions between gender, age, pubertal development, and stress on outcomes (Ho et al., 2012; Siddiqui & Romeo, 2019). It should also be noted that we did not observe significant differences in hippocampal macrostructure (i.e., MRI-based volumetric measures) between Irma-exposed and non-exposed group. While some developmental human imaging studies of stress have shown null volumetric alterations in the hippocampus (De Bellis et al., 1999; Tottenham et al., 2010) or even increases in hippocampal volume (Tupler & De Bellis, 2006; Woon & Hedges, 2008), others have demonstrated that traumatic events are associated with reductions in hippocampal volume (Lambert et al., 2017; Lee et al., 2018; McLaughlin et al., 2016, 2019; Weissman et al., 2020). That said, our current findings suggest that RSI may be a more sensitive measure than macrostructure measures of the hippocampus in the human brain. Future work is needed to further evaluate the relationship between RSI and other imaging modalities (e.g., sMRI, fMRI) to better understand how changes in RSI-based cellularity estimates relate to changes in brain structure and function. Lastly, there is speculation that the proportion of restricted diffusion estimated with RSI may also detect long cylindrical glial processes (White, Leergaard, et al., 2013). Given previous work showing microglial sensitivity to changes in environment (Walker et al., 2013), it is possible that the changes in hippocampal microstructure observed in the current study may also relate to changes in glial processes. However, current research on the response of glial cells to unpredictable stress is mixed (Delpech et al., 2016; Jauregui-Huerta et al., 2010; Paolicelli & Ferretti, 2017; Pearson-Leary et al., 2016) and more research is needed.

Overall, the present study provides novel evidence suggesting the potential utility of RSI in detecting behaviorally significant, yet subtle microstructure changes in the hippocampus in response to naturalistic stressful events. Although further research is needed, our results suggest that advanced diffusion MRI methodology may provide novel opportunities to elucidate how cumulative stressors and dimensions of stressors (e.g., threat vs. deprivation; McLaughlin et al., 2014), such as those experienced during the coronavirus disease 19 pandemic, impact youth. Given changing weather patterns and predicted increases in hurricanes and other natural disasters (Bender et al., 2010), our results provide important information that has relevance for researchers, clinicians, families, and policy makers for identifying and ultimately minimizing the impact of these events on young people.

## Supplementary Material

Supplemental materials

## Data Availability

Data used in the preparation of this article were obtained from the ABCD Study (https://abcdstudy.org), held in the NIMH Data Archive (NDA). This is a multisite, longitudinal study designed to recruit more than 10,000 children ages 9–10 and follow them over 10 years into early adulthood. The ABCD Study is supported by the National Institutes of Health and additional federal partners under award numbers U01DA041048, U01DA050989, U01DA051016, U01DA041022, U01DA051018, U01DA051037, U01DA050987, U01DA041174, U01DA041106, U01DA041117, U01DA041028, U01DA041134, U01DA050988, U01DA051039, U01DA041156, U01DA041025, U01DA041120, U01DA051038, U01DA041148, U01DA041093, U01DA041089, U24DA041123, and U24DA041147. A full list of supporters is available at https://abcdstudy.org/federalpartners.html. A listing of participating sites and a complete listing of the study investigators can be found at https://abcdstudy.org/consortium_members/. ABCD consortium investigators designed and implemented the study and/or provided data but did not necessarily participate in analysis or writing of this report. This manuscript reflects the views of the authors and may not reflect the opinions or views of the NIH or ABCD consortium investigators. The ABCD data repository grows and changes over time. The ABCD data used in this report came from https://doi.org/10.15154/1506087. DOIs can be found at nda.nih.gov/study.html?id=817.

## Footnotes

1 All main effects hold when including or excluding these participants. However, we focus on the sample with 25 participants randomly excluded in our main text to eliminate significant differences in age between the Irma-exposed and non-exposed groups.

2 Given previous work showing that development and hormonal changes during puberty can affect neuronal proliferation within the hippocampus (Allen et al., 2014; Hueston et al., 2017) and stress response (Viau, 2002), supplementary analyses using data from only pre- or early-pubertal participants was conducted and is provided in supplementary materials (Supplemental Analyses 1).

3 This result was consistent when including the 25 participants that were randomly excluded to reduce significant differences in age between the Irma-exposed and non-exposed groups, (***β*** = −4.20 × 10^−3^ (***SE*** = 1.34 × 10^−3^), ***t*** = −3.29, ***p*** = .001, ***r***^***2***^ (adj) = .05, Δ***r***^***2***^ (adj) = .02).

4 Ancillary analyses revealed no significant difference in hippocampal cellularity between groups scanned prior to and following Irma from the distal, Irma-non-exposed Northeastern site (***β*** = −1.67 × 10^−3^ (***SE*** = 1.31 × 10^−3^), ***t*** = 1.28, ***p*** = .20, ***r***^***2***^ (adj) = .03, Δ***r***^***2***^ (adj) < .001; Figure 6a).

5 Ancillary analysis revealed no significant difference in delayed recall between groups tested prior to and following Irma from the distal, non-exposed Northeastern site (***β*** = 0.06 (***SE*** = 0.35***), t*** = 0.187, ***p*** = .85, ***r***^***2***^ (adj) = .05, Δ***r***^***2***^ (adj) < .001; Figure 6b).
