# Supplementary material for: Altered hippocampal microstructure and function in children who experienced Hurricane Irma
Source: Dev Psychobiol. 2020 Dec 16;63(5):864–77. doi: 10.1002/dev.22071 (PMC8206237; doi:10.1002/dev.22071)
Supplement: Supplementary file 1 — Supplementary Material [file DEV-63-864-s001.zip › dev22071-sup-0004-Supinfo.docx]

Supplemental Materials

**Supplemental analyses 1: Pre- and early-pubertal participants**

Given previous work showing that gonadal hormonal changes that occur during puberty can affect hippocampal neurogenesis (Allen et al., 2014; Hueston et al., 2017) and stress response (Viau, 2002), we performed supplementary analyses identical to those presented in our main text using data from only pre- or early-pubertal participants from the South Florida site (non-exposed *n* = 129, Irma-exposed *n* = 142). Consistent with the findings presented in our main text, lower hippocampal cellularity was observed in the hippocampus in the Irma-exposed group relative to the non-exposed control group when tested in only pre/early-pubertal participants (𝛽 = -3.33 x 10^-3^(*SE* = 1.68 x 10^-3^*), t* = -1.98, *p* = .04, *r^2^* (adj) *=* .03, Δ*r^2^* (adj) = .01). Although this association does not withstand Bonferroni correction, a reduction in significance may be attributed to the reduction in sample size (Cohen, 1994; Royall, 1986) and it is notable that the magnitude and direction of the observed effect are consistent with the primary finding presented in our main text. Corresponding to the analyses in our main text, we next assessed the behavioral significance of any differences observed in hippocampal cellularity between the Irma-exposed and non-exposed groups by testing delayed recall memory performance. Consistent with the findings presented in our main text, and hypothesis 2, this analysis showed decreased delayed recall in the Irma-exposed group relative to the non-exposed control group when tested in only pre/early-pubertal participants (𝛽 = -0.87 (*SE* = 0.34*), t* = -2.54, *p* = .01, *r^2^* (adj) *=* .07, Δ*r^2^* (adj) = .02). This relationship was robust even when controlling for fluid intelligence (𝛽 = -0.82 (*SE* = 0.34*), t* = -2.41, *p* = .02, *r^2^* (adj) *=* .09, Δ*r^2^* (adj) = .02). Further, delayed recall was associated with hippocampal cell density (𝛽 = 24.53 (*SE* = 10.63*), t* = 2.307, *p* = .02, *r^2^* (adj) *=* .07, Δ*r^2^* (adj) = .01) with children with lower hippocampal cellularity showing poorer performance in delayed recall. Lastly, we evaluated whether changes in hippocampal cellularity were associated with delayed recall performance. This analysis was also consistent with the findings presented in our main text, revealing a significant association between hippocampal cellularity and delayed recall performance in the restricted pre/early-pubertal sample (𝛽 = 0.90 (*SE* = 0.34*), t* = -2.62, *p* < .01, *r^2^* (adj) *=* .07, Δ*r^2^* (adj) = .02). Overall, this analysis demonstrates that the associations presented in our primary text are robust to the known influences of puberty on changes in hippocampal neurogenesis and microstructure.

**Supplemental analyses 2: Parent-reported trauma history, threat exposure, and history of anxiety disorders and/or PTSD**

Exposure to adverse life events in childhood is common with some estimates indicating that nearly 50% of US children experience some form of adversity before age 18 (Sacks & Murphey, 2018). While the primary analyses presented in our main text focus on the exposure to the unpredictable event of Hurricane Irma on children in South Florida, it is likely that participants in both groups have histories of adverse life events. Adverse life events, and threatening events such as family conflict or neighborhood crime in particular, have previously been associated with changes in hippocampus such as reductions in volume (Lee et al., 2018; McLaughlin et al., 2016; McLaughlin et al., 2019) and activation during tasks (Lambert et al., 2017). Additionally, anxiety disorders and PTSD have also been associated with alterations in hippocampal volume (Gold et al., 2017; Mueller et al., 2013; Tupler and DeBellis, 2006; Woon and Hedges, 2008). Thus, additional analyses were performed controlling for parent-reported trauma history and history of anxiety disorders or PTSD and threat exposure in all primary models presented in our main text.

Trauma history was estimated by summing all items from the Parent Diagnostic Interview for DSM-5 (KSADS-5) Traumatic Events instrument to assign each participant a total count score (mean = 0.54 (*SD* = 1.42), range = 0-11) (Kobak et al., 2013). Events included in this instrument specifically include: *a car accident in which the child or another person in the car was hurt bad enough to require medical attention; another significant accident for which your child needed specialized and intensive medical treatment; witnessed or caught in a fire that caused significant property damage or personal injury; witnessed or caught in a natural disaster that caused significant property damage or personal injury; witnessed or present during an act of terrorism (e.g., Boston marathon bombing); witnessed death or mass destruction in a war zone; witnessed someone shot or stabbed in the community, shot, stabbed, or beaten brutally by a non-family member; shot, stabbed, or beaten brutally by a grown up in the home; beaten to the point of having bruises by a grown up in the home; a non-family member threatened to kill your child; a family member threatened to kill your child; witness the grownups in the home push, shove or hit one another; a grown up in the home touched your child in his or her privates, had your child touch their privates, or did other sexual things to your child; an adult outside your family touched your child in his or her privates, had your child touch their privates or did other sexual things to your child; a peer forced your child to do something sexually; and learned about the sudden unexpected death of a loved one.* This score was added as covariate to the models presented in our main text to evaluate whether observed effects were robust to trauma history.

History of anxiety disorders and/or PTSD was estimated using the Parent Diagnostic Interview for DSM-5 (KSADS-5) (Kobak et al., 2013). A binary variable was generated to indicate whether participants had any history of meeting the diagnostic criteria for separation anxiety, social anxiety, generalized anxiety, and/or PTSD. A total of 41 participants met criteria for a past anxiety disorder and/or PTSD. This variable was added as a covariate to the models presented in our main text to evaluate whether observed effects were robust to past anxiety disorders and/or PTSD.

Lastly, threat exposure (e.g., family conflict and neighborhood safety; McLaughlin et al., 2014) was estimated utilizing two variables from the ABCD Culture & Environment battery previously described in Zucker and colleagues (2018). Family conflict was measured using the youth-report summary score for the Conflict Subscale from the Family Environment Scale (mean= 2.98 (*SD* = 1.81), range= 1-10; Moos and Moos, 1994) and neighborhood crime was measured using a composite score generated by averaging the ABCD Youth Neighborhood Safety/Crime Survey and ABCD Parent Neighborhood Safety/Crime Survey (both modified from PhenX; mean= 3.92 (*SD* = 0.80), range= 1.5-5; Echeverria et al., 2004; Mujahid et al., 2007). Both of these summary scores were separately added as covariates to the models presented in our main text to evaluate whether observed effects were robust to these forms of threat exposure.

Consistent with the findings presented in our main text, lower hippocampal cellularity was observed in the hippocampus in the Irma-exposed group relative to the non-exposed control group when controlling for trauma history, past anxiety disorders and/or PTSD, and threat exposure (𝛽 = -3.51 x 10^-3^(*SE* = 1.42 x 10^-3^*), t* = -2.47, *p* = 0.01, *r^2^* (adj) *=* .04, Δ *r^2^* (adj) = .02). Additionally, decreased delayed recall in the Irma-exposed group relative to the non-exposed control group also remained robust when controlling for trauma history, past anxiety disorders and/or PTSD, and threat exposure (𝛽 = -0.66 (*SE* = 0.29*), t* = -2.28, *p* = .02, *r^2^*(adj) *=* .12, Δ*r^2^*(adj) = .01). Next, we evaluated whether changes in hippocampal cellularity were associated with delayed recall performance when controlling for trauma history, past anxiety disorders and/or PTSD, and threat exposure. Corresponding to our primary findings, delayed recall was associated with hippocampal cellularity (𝛽 = 25.77 (*SE* = 10.92*), t* = 2.36, *p* = .02, *r^2^* (adj) *=* .07, Δ*r^2^* (adj) = .01) even when controlling for trauma history, past anxiety disorders and/or PTSD, and threat exposure. Overall, this supplemental analysis further supports the associations presented in our primary text and demonstrates that these findings are robust to the impact of various adverse life events or experiences on hippocampal microstructure.

**References**

Cohen, J. (1994). The earth is round (p<. 05). *American psychologist*, *49*(12), 997.

Gold, A. L., Steuber, E. R., White, L. K., Pacheco, J., Sachs, J. F., Pagliaccio, D., Berman, E., Leibenluft, E., & Pine, D. S. (2017). Cortical Thickness and Subcortical Gray Matter Volume in Pediatric Anxiety Disorders. Neuropsychopharmacology, 42(12), 2423–2433. <https://doi.org/10.1038/npp.2017.83>

Echeverria, S. E., Diez-Roux, A. V., et al. (2004) Reliability of self-reported neighborhood characteristics. J Urban Health 81(4): 682-701

Kobak, K. A., Kratochvil, C. J., Stanger, C., & Kaufman, J. (2013). Computerized screening of comorbidity in adolescents with substance or psychiatric disorders. *Anxiety Disorders and Depression.(La Jolaa, CA)*.

Lambert, H. K., Sheridan, M. A., Sambrook, K., Rosen, M., Askren, M. K., & McLaughlin, K. A. (2017). Hippocampal Contribution to Context Encoding across Development is Disrupted following Early-Life Adversity. Journal of Neuroscience, 2618–16. https://doi.org/10.1523/JNEUROSCI.2618-16.2017

Lee, S. W., Yoo, J. H., Kim, K. W., Kim, D., Park, H., Choi, J., Teicher, M. H., & Jeong, B. (2018). Hippocampal Subfields Volume Reduction in High Schoolers with Previous Verbal Abuse Experiences. Clinical Psychopharmacology and Neuroscience, 16(1), 46–56. <https://doi.org/10.9758/cpn.2018.16.1.46>

McLaughlin, K. A., Sheridan, M. A., & Lambert, H. K. (2014). Childhood adversity and neural development: deprivation and threat as distinct dimensions of early experience. *Neuroscience & Biobehavioral Reviews*, *47*, 578-591.

McLaughlin, K. A., Sheridan, M. A., Gold, A. L., Duys, A., Lambert, H. K., Peverill, M., Heleniak, C., Shechner, T., Wojcieszak, Z., & Pine, D. S. (2016). Maltreatment Exposure, Brain Structure, and Fear Conditioning in Children and Adolescents. Neuropsychopharmacology, 41(8), 1956–1964. https://doi.org/10.1038/npp.2015.365

McLaughlin, K. A., Weissman, D., & Bitrán, D. (2019). Childhood Adversity and Neural Development: A Systematic Review. Annual Review of Developmental Psychology, 1(1), 277–312. https://doi.org/10.1146/annurev-devpsych-121318-084950

Moos, R. H., and Moos, B. S. (1994) Family Environment Scale manual (3rd ed.). Palo Alto, CA: Consulting Psychologists Press.

Mueller, S. C., Aouidad, A., Gorodetsky, E., Goldman, D., Pine, D. S., & Ernst, M. (2013). Grey Matter Volume in Adolescent Anxiety: An Impact of the Brain-Derived Neurotropic Factor Val66Met Polymorphism? Journal of the American Academy of Child and Adolescent Psychiatry, 52(2), 184–195. https://doi.org/10.1016/j.jaac.2012.11.016

Mujahid, M. S., et al. (2007) Assessing the measurement properties of neighborhood scales: from psychometrics to ecometrics. Am J Epidemiol 165(8): 858-67.

Royall, R. M. (1986). The effect of sample size on the meaning of significance tests. *The American Statistician*, *40*(4), 313-315.

Sacks, V., & Murphey, D. (2018). The prevalence of adverse childhood experiences, nationally, by state, and by race or ethnicity. <https://www.childtrends.org/publications/prevalence-adverse-childhood-experiences-nationally-state-race-ethnicity>. Updated February 20, 2018. Accessed August 21, 2020.

Zucker, R. A., Gonzalez, R., Ewing, S. W. F., Paulus, M. P., Arroyo, J., Fuligni, A., ... & Wills, T. (2018). Assessment of culture and environment in the Adolescent Brain and Cognitive Development Study: Rationale, description of measures, and early data. *Developmental cognitive neuroscience*, *32*, 107-120.


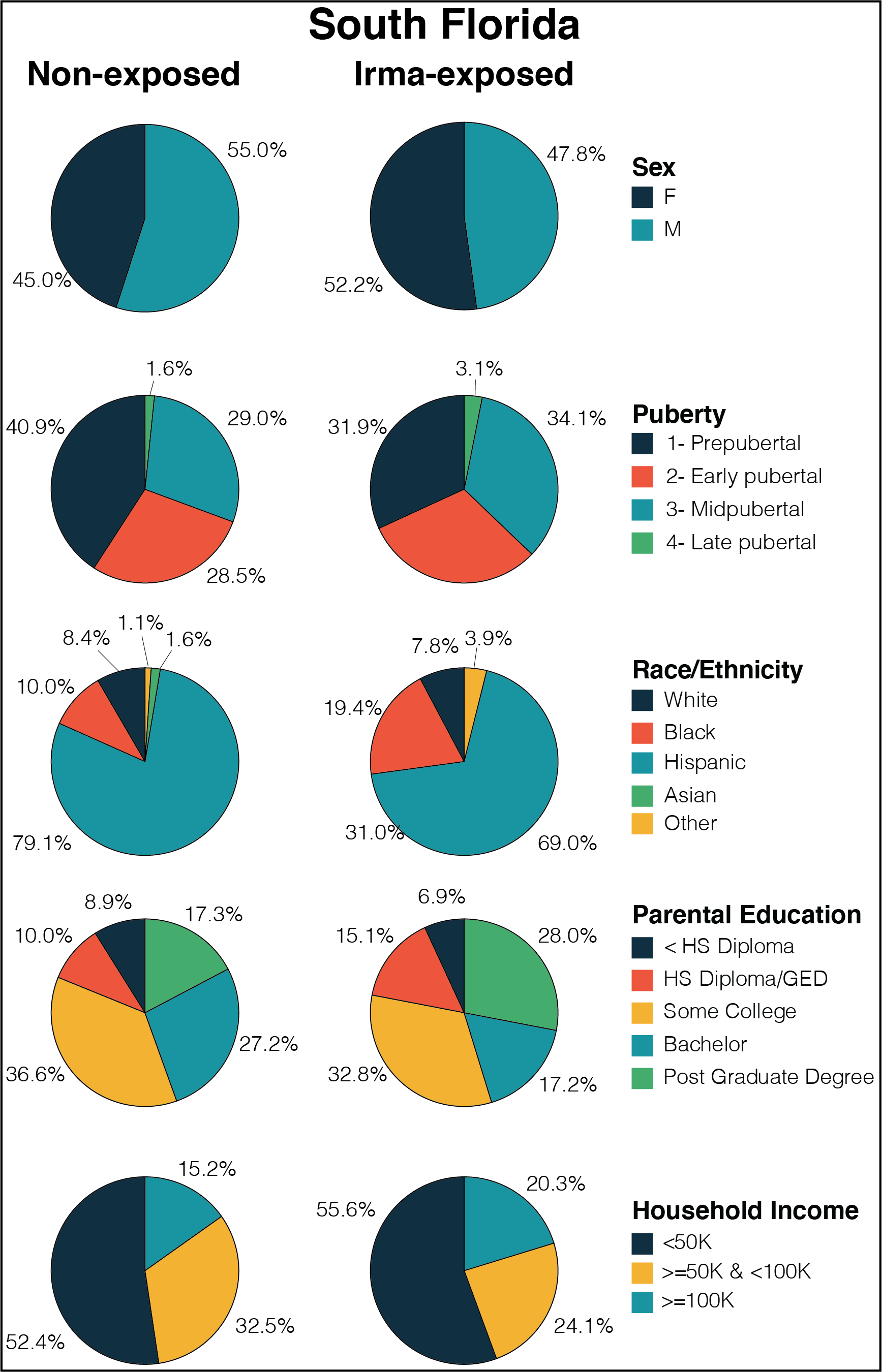


**Figure S1**. **Demographic variable distribution between non-exposed and Irma-exposed groups from the Southern Florida site.**

**
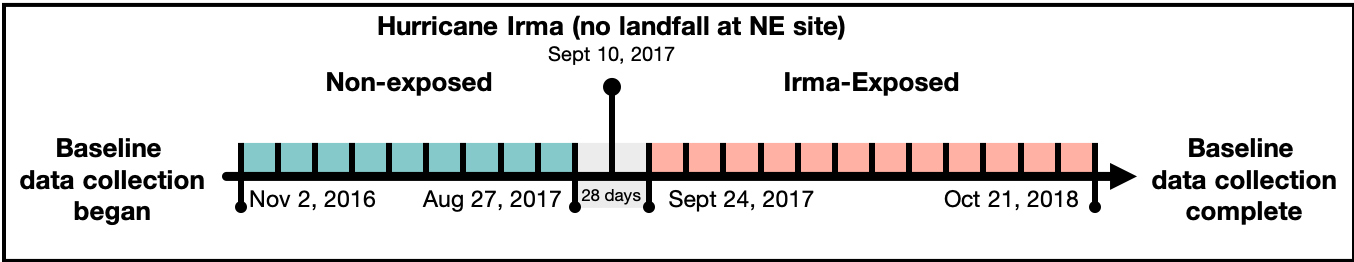
**

**Figure S2**. **Experimental design**. Participants from the distal, non-exposed Northeastern site were divided into 2 groups based on when baseline assessments and scans were acquired. The Pre-Irma group was scanned during the year prior to Hurricane Irma. The Post-Irma group was scanned during the year following Hurricane Irma.

**
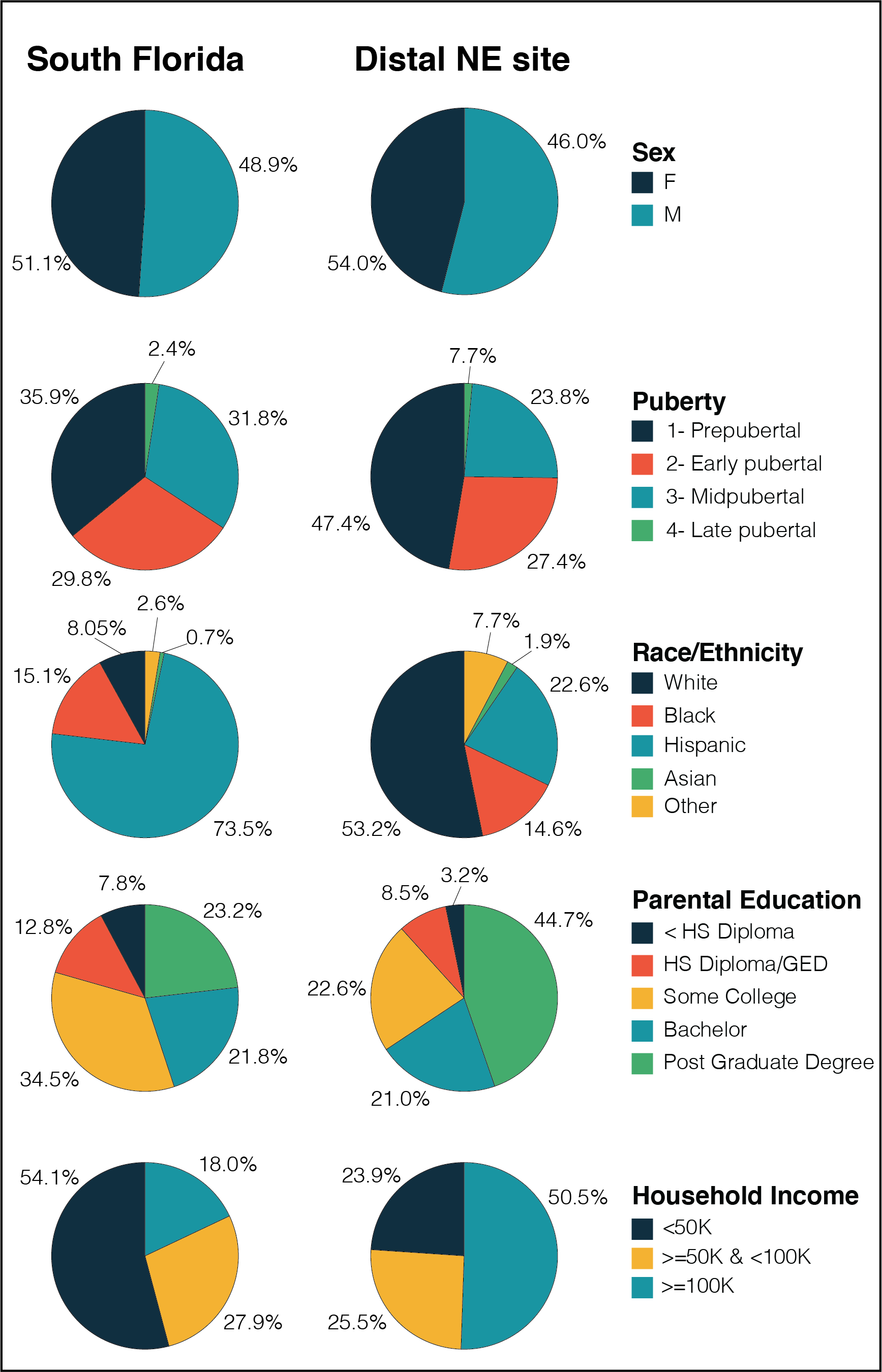
**

**Figure S3.** **Demographic variable distribution between South Florida and distal, non-exposed Northeastern site.**
