# Supplementary figures and images for: Altered hippocampal microstructure and function in children who experienced Hurricane Irma
Source: Dev Psychobiol. 2020 Dec 16;63(5):864–77. doi: 10.1002/dev.22071 (PMC8206237; doi:10.1002/dev.22071)

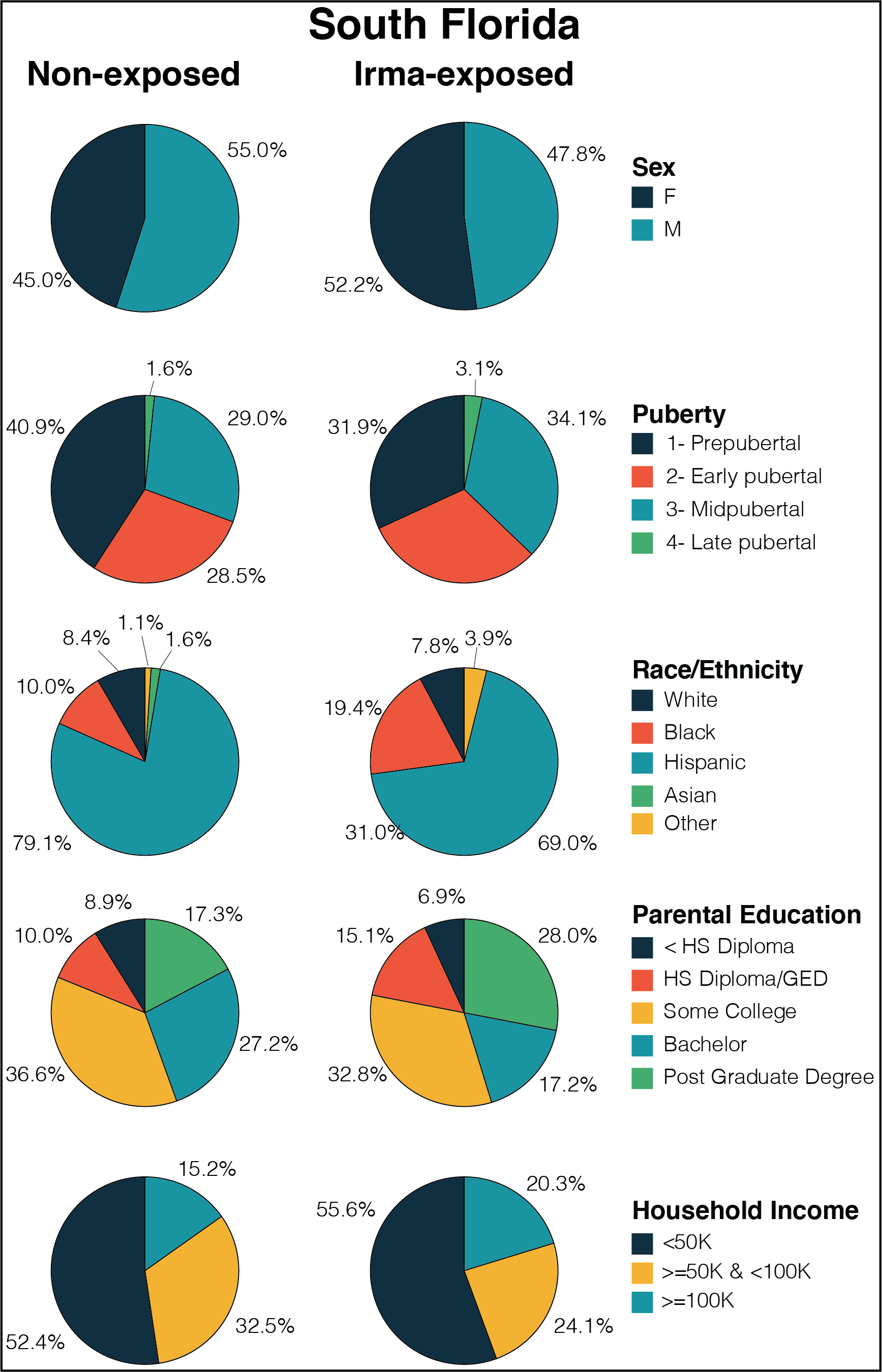

Supplement: Supplementary file 1 — Supplementary Material [file DEV-63-864-s001.zip › dev22071-sup-0001-FigureS1.png]

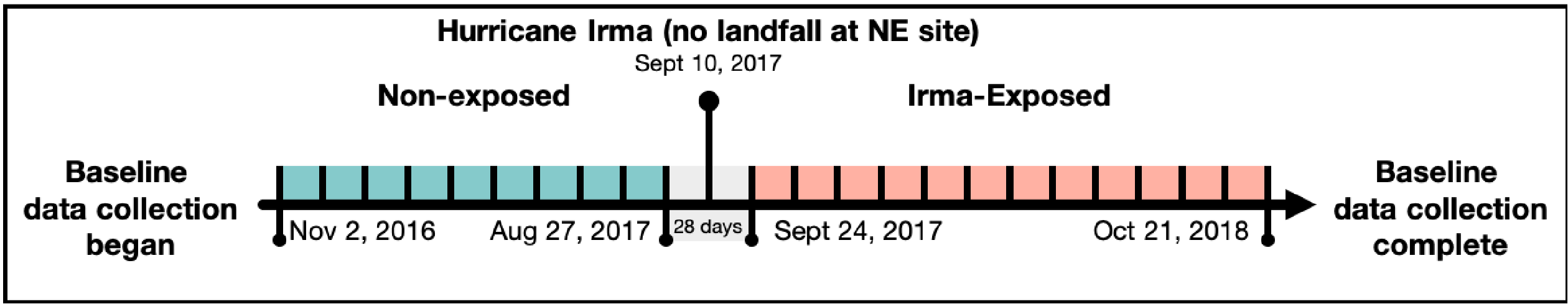

Supplement: Supplementary file 1 — Supplementary Material [file DEV-63-864-s001.zip › dev22071-sup-0002-FigureS2.png]

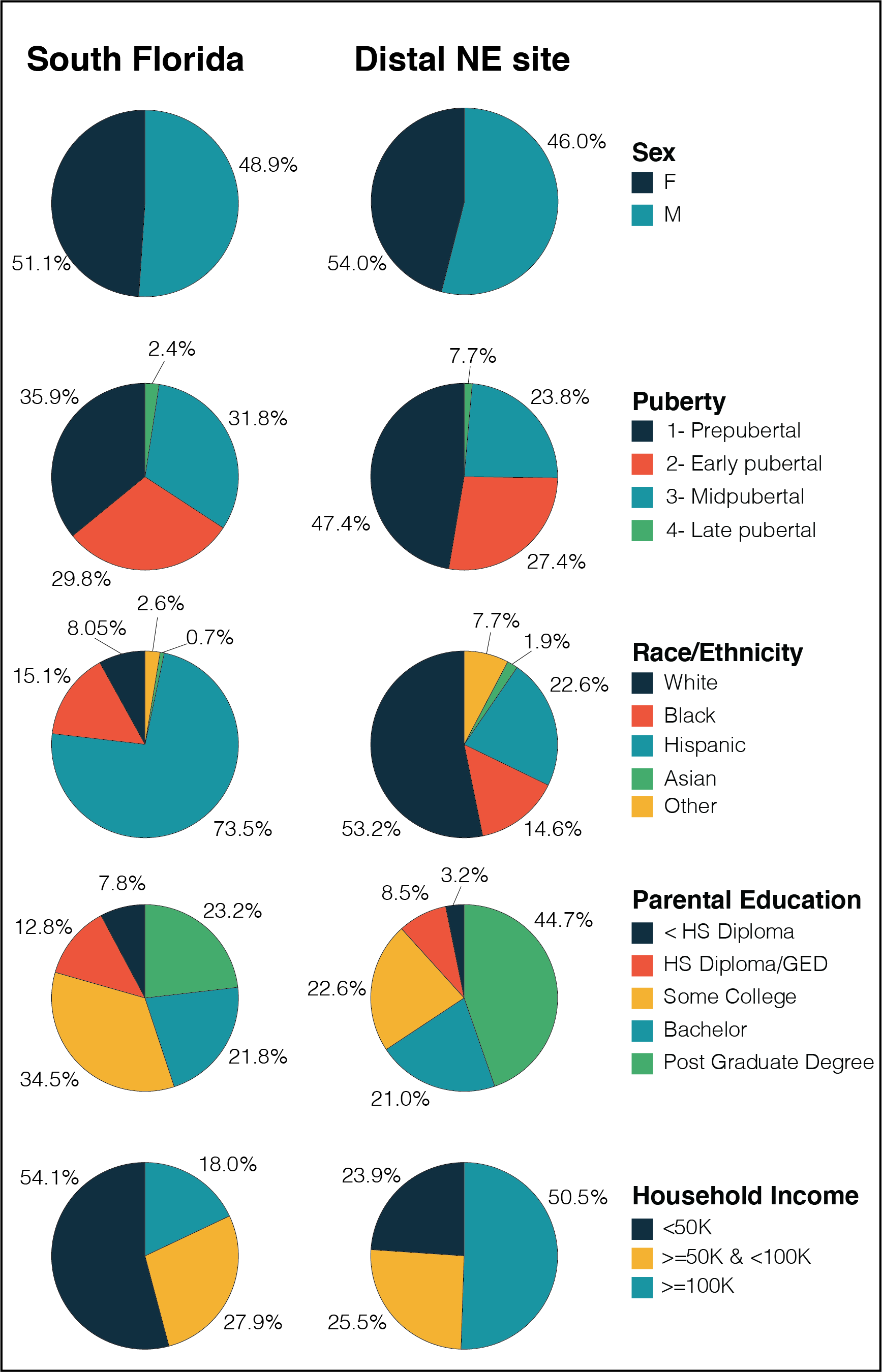

Supplement: Supplementary file 1 — Supplementary Material [file DEV-63-864-s001.zip › dev22071-sup-0003-FigureS3.png]
